# International Principles of Deceased Donor Organ Allocation

**Published:** 2013-05-01

**Authors:** Francis L. Delmonico

**Affiliations:** Professor of Surgery, Harvard Medical School, Massachusetts General Hospital, Transplant Center, Boston, MA 02114–2696, USA, Tel: +1-617-726-2825, E-mail: francis_delmonico@neob.org

Transplant professionals are entrusted with a unique position in the practice of medicine—the stewardship of the organs from either the deceased or living donor. This stewardship entails two major responsibilities for which society holds transplant professionals accountable: the equitable allocation of deceased donor organs to medically suitable recipients, and the evaluation and care for the living organ donor. Data, experience and ethical principles relevant to organ transplantation become invaluable in fulfilling these responsibilities and they are presented in this Editorial.

**The Declaration of Istanbul**

The policies regarding the allocation of organs now have reference guides published in the medical literature as a resource for practicing transplant professionals. For example, the Declaration of Istanbul emphasizes that organs for transplantation should be equitably allocated within countries or jurisdictions to suitable recipients regardless of gender, ethnicity, religion, social or financial status. Financial considerations or material gain of any party must not influence the application of relevant allocation rules.

**WHO Guiding Principles**

The Declaration of Istanbul was followed by the development of guidelines of practice by the WHO that also address the allocation of organs. Guiding Principle 9 states that “the allocation of organs, cells and tissues should be guided by clinical criteria and ethical norms, not financial or other considerations. Allocation rules defined by properly constituted committees should be equitable, externally justified, and transparent.”

**The Final Rule Implemented by the United Network for Organ Sharing (UNOS)**

A framework termed the Final Rule for the structure and operation of the Organ Procurement Transplant Network (OPTN) in the United States has been contracted to a private organization, the United Network for Organ Sharing (UNOS). The OPTN Final Rule gives the OPTN Board of Directors responsibility for developing policies for the OPTN in specific areas:

- Equitable allocation of deceased donor organs,
- Testing of organ donors and follow-up of transplant recipients to prevent the spread of infectious diseases,
- Reducing inequities resulting from socioeconomic status, and
- The training and experience of transplant surgeons and transplant physicians in designated transplant programs.

The Department of Health and Human Services directed the OPTN/UNOS to set priorities for ranking candidates on the waitlist based upon measurable medical criteria and ordered by medical need. Section 121.8 of the Final Rule addresses the allocation of organs and states that allocation policies: The allocation

- Shall be based upon sound medical judgment,
- Shall seek to achieve the best use of donated organs,
- Shall preserve the ability of a transplant program to decline an offer for a specific potential recipient,
- Shall be designed to avoid wasting organs, avoid futile transplants and promote patient access to transplantation and efficient management of organ placement,
- Shall not be based upon the candidate’s place of residence or place of listing.

**Allocation Performance Goals and Assessment of Performance**

The OPTN Final Rule includes performance goals for allocation policies. In addition to the use of objective and measurable medical criteria, the policies provide for candidate rankings that give priority to patients based upon medical urgency with patients having the most urgent need receiving highest priority. Each organ-specific allocation policy should include performance indicators, determining by data on how closely the results of the current allocation policy approach the performance goals; and thus, assure the public that ethical principles are being upheld.

An important factor for the OPTN in its policy development deliberation, is the fact that the National Organ Transplant Act (NOTA) limits the policy considerations for organ allocation policy to medical criteria. The Final Rule amplifies this restriction by requiring that the patient rankings, resulting from the allocation policy, be based upon objective and measurable medical criteria. Therefore, allocation policies do not take into account social criteria such as social worth or economic criteria. Organ allocation policy does not consider, for example, whether a patient is an unemployed vagrant as opposed to the CEO of a major corporation. Likewise, the underlying cause of a disease such as drug or alcohol addiction is not considered by the allocation policies. An individual transplant program may consider whether a patient has maintained a suitable period of abstinence as part of its evaluation of whether the patient is an acceptable candidate, but once a patient is on the OPTN waiting list, whether the patient’s disease was caused by substance abuse may not be considered because it is understood to be “social criteria.”

**Application of Ethical Principles**

In the setting of the Final Rule, classic ethical principles of justice, utility, benefits, and non-maleficence and autonomy become evident. The Final Rule accounts for the justice principle by requiring allocation policy to be based upon sound medical judgment. Justice calls for the distribution of kidneys to patients with the longest waiting time. Ultimately, all patients with the same medical condition should be treated the same when placed on the waitlist. For extra-renal organs, the justice principle now mandates medical need or the severity of the patient’s illness as the foremost criterion of allocation. Utility**,** as an ethical principle of the Final Rule, seeks the best use of the donated organ in achieving the best allograft survival; the best outcome. Beneficence promotes the interest of the patient to undergo transplantation. The Final Rule sustains non-maleficence “to do no harm” by enabling a transplant center to decline a specific organ for a specific patient. Finally, autonomy preserves the ultimate decision of the patient to undergo transplantation. 

**The Development of a Waitlist**

Following these principles, local policies for organ allocation should be developed for patients on a waiting list that is applied with justice, utility and equity. Under its OPTN contract with Health Resources and Services Administration (HRSA), UNOS maintains a centralized computer network linking all organ procurement organizations and transplant centers. This computer network is accessible 24 hours a day, seven days a week. When a donated organ from a deceased donor is available, the Organ Procurement Organization (OPO) must enter information about the donor into the OPTN contractor’s computer system and execute the computer match program. That program will rank order the candidates on the OPTN waiting list according to the organ allocation policies that have been adopted by the Board of Directors. Each patient in the database is matched by the computer against the donor characteristics. The computer then generates a ranked list of candidates for each available organ in ranked order according to OPTN organ allocation policies. The OPO then offers the organ to designated transplant programs in accordance to the rank order of the match. The transplant program may accept or refuse the offer. The match is patient-specific as required by NOTA. The organ may not simply be offered to a transplant program for the program to use at its discretion. If an organ is offered to a transplant program for a certain patient, but the program decides to decline the offer, the organ is then offered to the next patient on the ranked OPTN match list even if the patient is at another institution. 

**Eurotransplant**

Eurotransplant is responsible for the allocation of donor organs in Austria, Belgium, Croatia, Germany, Luxembourg, the Netherlands, and Slovenia. This international collaborative framework includes all transplant hospitals, tissue-typing laboratories and hospitals where organ donations take place. Eurotransplant was one of the first international organizations founded by Prof. Jon J. van Rood in 1967. The following is recorded at the Eurotransplant Web site: all transplantation centers within the member states of Eurotransplant have access to the central computer database. In this database, the transplantation centers enter the general and medical information of their recipients along with the recipient profile and the donor profile. As soon as a donor becomes available within the Eurotransplant area, the regional tissue-typing laboratory determines the donor’s blood group and tissue characteristics. Eurotransplant generates a so-called “match list” for each donor organ, which in effect addresses a single waitlist of patients within the serviced countries. 

The match list is generated by a computer algorithm that takes into account all medical and ethical criteria and is based upon the expected outcome and the medical urgency of a certain patient.

The allocation system of Eurotransplant has the following attributes:

- Objective: the match list is the same no matter which duty desk officer arranges the allocation.
- Reproducible: the same question will lead to the same answer.
- Transparent: every step in the process can be accounted for. And,
- Valid: the system is based upon valid medical and ethical criteria that are supported by consensus within the transplant community.

**Solicitation for Organ Donors**

Another important principle of the WHO that was adopted by the 63^rd^ World Health Assembly in 2010 addresses solicitation of organs in the background of ethically proper allocation. Guiding principle 6 states that “advertising the need for or availability of cells, tissues or organs, with a view to offering or seeking payment to individuals for their cells, tissues or organs or to the next of kin where the individual is deceased, should be prohibited. Brokering that involves payment to such individuals or to 3^rd^ parties should also be prohibited.” The WHO goes on to state further “that this principle does not affect general advertisements or public appeals to encourage altruistic donation, provided they do not subvert legally establish systems of organ donation.” In the United States, and elsewhere in the world, there have been billboards and media advertisements by those in need of transplants attempting to bring public attention to their plight. UNOS has develop an important policy regarding billboard advertisements for deceased donors, the way an individual may establish a relationship with a potential living donor, and the opportunity for directed donation when deceased donor allocation is considered. 

UNOS makes a distinction between solicitations for live donor organ *vs*. solicitations for directed donation of deceased organs. Solicitation for live donor organs is well known and pleas are being made today via the Internet. These Internet interactions are a matter of personal choice to convey medical and social information to potential vast audience in hopes of receiving a transplant. Aside from the Internet, appeals can also be made in a variety of other ways that enable personal relationship develop and result in the donation of an organ from a living person to a specific recipient. Regulatory authorities should not restrict the ways relationships are developed with respect to live organ donation while upholding the provisions of WHO standards that it is unethical and widely illegal for any person to acquire a human organ for transplantation on the basis of monetary compensation to a vendor. Transplant centers also have a responsibility in helping protect potential recipients from the hazards that can arise from public appeals. The psychosocial evaluation of live donors should assess the motivation of these individuals to make certain that they will not be imposing upon the organ transplant recipient for the duration of their life.

Regarding public solicitation for deceased donor organs, regulatory authorities should establish policy regarding the appropriateness of donor-donee relationship in directed donations from deceased donors. Any relationship forms via public or commercial solicitation, for example by the use of billboards will undermine the public trust in the allocation system if certain patients are advantaged to receive the next available organ because of such commercial solicitation.

**Directed Donation**

By my personal experience as medical director of the New England Organ Bank (NEOB), and consistent with the policy of the OPTN/UNOS regarding directed donation, the NEOB has participated in facilitating deceased organ donation by a person authorized to consented donation when the following conditions are present:

- The donate in the directed donation is a specific named individual.
- There is no monetary exchange of valuable consideration or other coercive inducement involved in the donation. And,
- When relevant law of a jurisdiction permits such directed donation (consistent with WHO guidelines).

The NEOB does not participate in donations which discriminate against the person or class of persons on the basis of race, national origin, religion, gender or any other such social characteristics. The NEOB contacts the appropriate transplant center to describe the opportunity of directed donation from a specific donor intended for a specific potential recipient to determine medical suitability and medical preparedness to undergo transplantation. 

**The Public Trust**

The public trust is undermined when there is a violation of ethical principles in the implementation of the organ allocation system. Society will not donate if it has the impression that the national system of organ donation is corrupt. Media reports of such instances become the source of concern and require societal attention as exercise through appropriate regulatory authority such as the Ministry of Health, *etc*. 

Organ allocation becomes a testimony of social justice within a country or jurisdiction. No culture of the world rejects the opportunity of medical treatment by organ transplantation. No culture of the world should then reject organ donation from the deceased. Conversely, no society can sustain a system of deceased organ donation if those that donate cannot be recipients within that country or jurisdiction. 

The WHO has promulgated the most important concluding guideline of transparency, *i.e.*, “the organization and execution of donation and transplantation activities, as well as their clinical results must be transparent and open to scrutiny, while ensuring that personal anonymity and privacy of donors and recipients are always protected.”

